# Galectin-9, a Player in Cytokine Release Syndrome and a Surrogate Diagnostic Biomarker in SARS-CoV-2 Infection

**DOI:** 10.1128/mBio.00384-21

**Published:** 2021-05-04

**Authors:** Najmeh Bozorgmehr, Siavash Mashhouri, Eliana Perez Rosero, Lai Xu, Shima Shahbaz, Wendy Sligl, Mohammed Osman, Demetrios J. Kutsogiannis, Erika MacIntyre, Conar R. O’Neil, Shokrollah Elahi

**Affiliations:** a School of Dentistry, Division of Foundational Sciences, University of Alberta, Edmonton, Alberta, Canada; b Department of Medicine, University of Alberta, Edmonton, Alberta, Canada; c Department of Critical Care Medicine, University of Alberta, Edmonton, Alberta, Canada; d Division of Infectious Diseases, Faculty of Medicine and Dentistry, University of Alberta, Edmonton, Alberta, Canada; e Misericordia Community Hospital, Edmonton, Alberta, Canada; f Department of Medical Microbiology and Immunology, University of Alberta, Edmonton, Alberta, Canada; g Department of Medical Oncology, University of Alberta, Edmonton, Alberta, Canada; h Li Ka Shing Institute of Virology, Faculty of Medicine and Dentistry, University of Alberta, Edmonton, Alberta, Canada; Medical School, National and Kapodistrian University of Athens

**Keywords:** COVID-19, Galectin-9, cytokines, monocytes, SARS-CoV-2, cytokine storm, NK cells, chemokines, neutrophils

## Abstract

The outbreak of SARS-CoV-2 infection has enormously impacted our lives. Clinical evidence has implicated the emergence of cytokine release syndrome as the prominent cause of mortality in COVID-19 patients. In this study, we observed massive elevation of plasma Galectin-9 (Gal-9) in COVID-19 patients compared to healthy controls (HCs). By using the receiver operating characteristic (ROC) curve, we found that a baseline of 2,042 pg/ml plasma Gal-9 can differentiate SARS-CoV-2-infected from noninfected individuals with high specificity/sensitivity (95%). Analysis of 30 cytokines and chemokines detected a positive correlation of the plasma Gal-9 with C-reactive protein (CRP) and proinflammatory cytokines/chemokines such as interleukin-6 (IL-6), tumor necrosis factor alpha (TNF-α), IP-10, MIP-1α, and MCP-1 but an inverse correlation with transforming growth factor β (TGF-β) in COVID-19 patients. In agreement, we found enhanced production of IL-6 and TNF-α by monocytes and NK cells of COVID-19 patients once treated with the recombinant human Gal-9 *in vitro*. Also, we observed that although the cell-membrane expression of Gal-9 on monocytes does not change in COVID-19 patients, those with higher Gal-9 expression exhibit an activated phenotype. Furthermore, we noted significant downregulation of surface Gal-9 in neutrophils from COVID-19 patients compared to HCs. Our further investigations indicated that immune activation following SARS-CoV-2 infection results in Gal-9 shedding from neutrophils. The strong correlation of Gal-9 with proinflammatory mediators suggests that inhibition of Gal-9 may severe as a therapeutic approach in COVID-19 infection. Besides, the plasma Gal-9 measurement may be used as a surrogate diagnostic biomarker in COVID-19 patients.

## INTRODUCTION

The severe acute respiratory syndrome coronavirus-2 (SARS-CoV-2) has enormously impacted the world since December 2019. It presents with a variety of clinical symptoms collectively defined as coronavirus 2019 disease (COVID-19) (1). Although most infected individuals remain asymptomatic or present with mild symptoms, some become seriously ill and require hospitalization (2). Among patients admitted to hospitals, acute respiratory distress syndrome (ARDS) is a common complication, which can result in multiorgan failure and death (3). Although the mechanism of lung injury and multiorgan failure in COVID-19 is not well understood (4), the cytokine storm may contribute to the pathogenesis of COVID-19 infection (5, 6). In addition to the direct effects of the virus on immune and nonimmune cells, a complex interconnected network of cells, signaling pathways, and soluble mediators can contribute to the induction of the cytokine storm (7).

Innate immune cells, by way of pattern recognition receptors (PRRs) or damage recognition receptors (DRRs), recognize and respond to a wide variety of pathogens (e.g., by cytokine and chemokine secretion) (8). For example, activated macrophages/monocytes secrete excessive amounts of cytokines including gamma interferon (IFN-γ), interleukin-1 (IL-1), IL-6, tumor necrosis factor alpha (TNF-α), and IL-18 (9). Similarly, neutrophils produce neutrophil extracellular traps and natural killer (NK) cells prolong the antigenic stimulation due to diminished/skewed cytolytic functions, both of which can amplify cytokine production (10). Overall, it appears that the viral burden (11), accompanied by the dysregulated innate immune response (12) due to underlying conditions (13), and aging (14) may ignite the cytokine storm. The “cytokine release syndrome” has been used to clinically define the higher mortality risk in COVID patients (15). Current research has suggested that the extensive release of endogenous damage-associated molecular pattern (DAMP) molecules (such as HMGB1, etc.) exacerbates the disease (16, 17).

Galectin-9 (Gal-9) is a tandem repeat galectin (18) which binds to carbohydrates and proteins and is widely expressed in the nucleus, cytosol, and outer plasma membrane and extracellular matrix (19). Gal-9, like other galectins, is synthesized on free polysomes in the cytoplasm and secreted through nonclassical pathways or released freely upon cell death. Some galectins are produced by immune cells, whereas others are produced by nonimmune cells (e.g., epithelial or endothelial cells) (20). Galectins are involved in many biological functions such as development, signal transduction, and immune responses (21). Gal-9 mediates different biological properties, which depends on its receptor and the source (e.g., extracellular, intracellular, or membrane bound) (22–25).

Gal-9 upon interaction with its receptors such as TIM-3, protein disulfide isomerase (PDI), 4-1BB, IgE, CD44, and Dectin-1 exhibits distinct and sometimes opposing effects (19, 22, 26, 27). For example, it induces chemotaxis, activates eosinophils, and enhances cytokine secretion in mast cells (28). Besides, it promotes monocyte-derived dendritic cell (DC) maturation (29) and enhances the function of DCs, and NK cells via facilitating 4-1BB signaling (30). Recently, the potential role of galectins as PRRs has become an area of intense research (31, 32). Also, galectins have been considered DAMPs that alongside the pathogen-associated molecular patterns (PAMPs) orchestrate the innate immune response (33). For example, Gal-9 is reported as a danger signal in dengue virus infection (34). Similarly, the role of Gal-3 in the induction of cytokine storm has been reported (35). Subsequently, the potential role of Gal-3 inhibitors as a therapeutic approach to attenuate the hyperinflammation in COVID-19 infection has been suggested (36). However, the role of Gal-9 in COVID-19 infection has not been well studied, although its upregulation has been reported (37). Therefore, we decided to elucidate the role of Gal-9 in COVID-19 infection. In particular, we measured Gal-9 concentrations in COVID-19 patients versus healthy controls (HCs) and in comparison with HIV-infected individuals and patients with virus-associated solid tumors.

## RESULTS

### The plasma Gal-9 level discriminates COVID-19 patients from healthy subjects.

**FIG 1:**
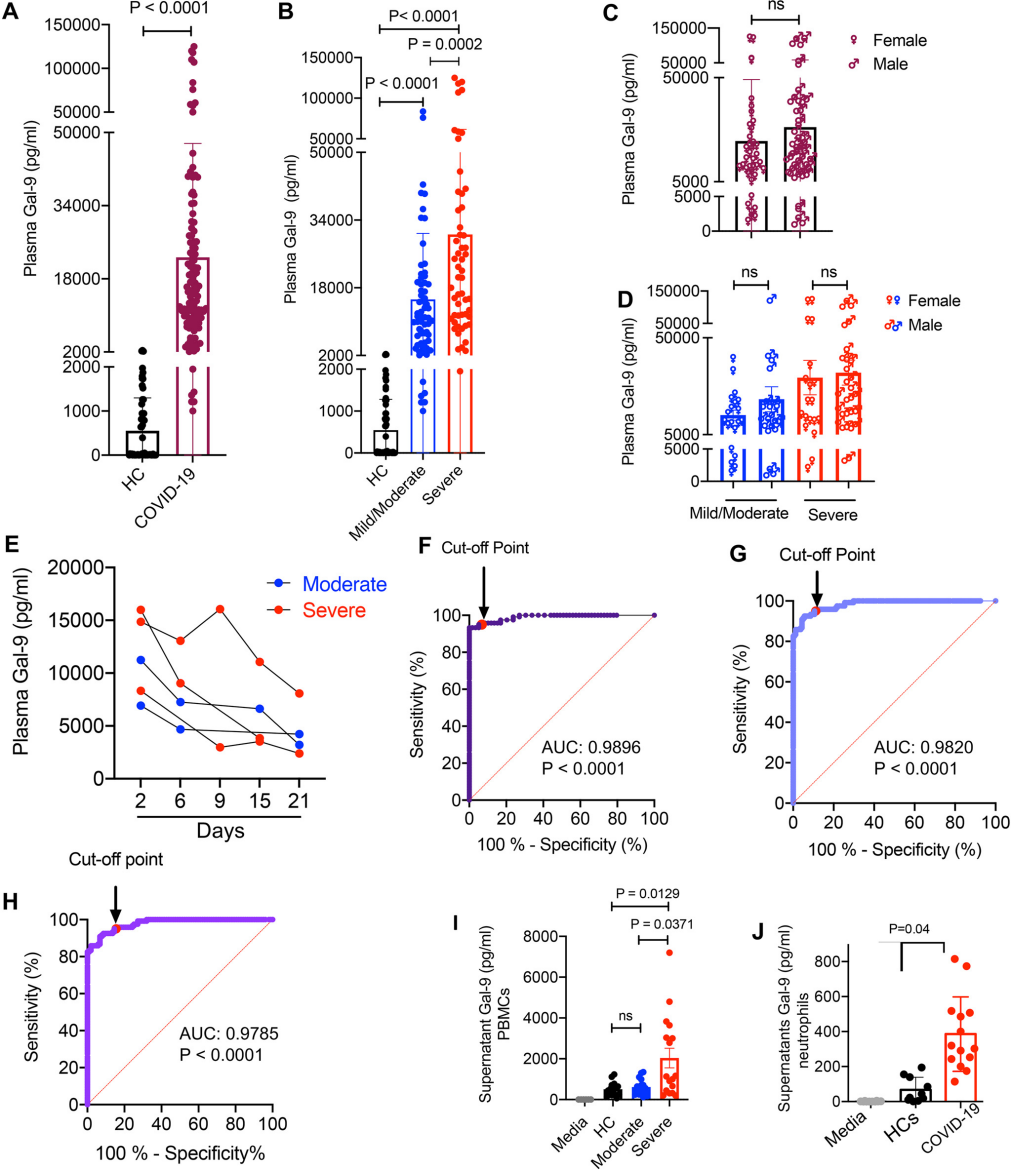
The massive elevation of plasma Gal-9 discriminates COVID-19 patients from healthy subjects. (A) Detected concentrations of Gal-9 in the plasma specimens of COVID-19-infected individuals compared to healthy controls (HCs) as measured by ELISA. (B) Detected plasma levels of Gal-9 in COVID-19 patients with mild/moderate or severe disease versus HCs. (C and D) Comparing the plasma levels of Gal-9 in total COVID-19-infected females versus males (C) and those with mild/moderate versus severe disease (D). (E) Graph showing the plasma Gal-9 levels in two COVID-19 patients with moderate and two with severe disease 1 day posthospitalization up to 21 days later. (F) The receiver operating characteristic (ROC) curve of comparison between plasma Gal-9 levels in COVID-19 patients (*n* = 120) and HCs (*n* = 57). (G) The ROC curve of comparison of plasma Gal-9 levels in COVID-19 patients (*n* = 120) versus HCs + cancer patients + HIV-infected individuals (*n* = 161). (H) The ROC curve of comparison of plasma Gal-9 levels in COVID-19 patients (*n* = 120) versus cancer patients + HIV-infected individuals (*n* = 103). (I and J) Detected concentration of Gal-9 in culture supernatants of PBMCs (I) and neutrophils (J) from COVID-19 patients or HCs after 6 h of incubation at 37°C as quantified by ELISA. Each point represents data from a patient. Bar, mean ± 1 standard error. AUC, area under curve; ns, not significant.

The elevation of Gal-9 in the plasma of patients with viral infections such as HIV infection (24), virus-associated carcinoma (25), hepatitis B and C, and dengue fever (19, 38) has been well documented. Therefore, we decided to determine if this was the case for COVID-19 infection. We measured the plasma Gal-9 in a cohort of 120 SARS-CoV-2-infected individuals and 59 healthy controls (HCs). We found significantly higher levels of Gal-9 in the plasma of COVID-19 patients compared to HCs (ranging between 0 and 2,042 pg/ml) (Fig. 1A). After dividing the patients based on their clinical status (mild/moderate versus severe as defined in Materials and Methods), we observed that plasma Gal-9 concentrations were substantially greater in those with severe disease (ranging between 1,950 and 125,510 pg/ml) compared to those with mild/moderate disease (ranging between 1,000 and 83,717 pg/ml) (Fig. 1B). Next, we compared the plasma Gal-9 in males versus females, but we did not find any significant difference in the plasma Gal-9 concentration between total males and females (Fig. 1C) or even when subdivided based on their clinical disease presentation (Fig. 1D). Also, we were able to collect blood for some patients at different time points to track the plasma Gal-9 pattern during a timeline up to 21 days. These results indicated a gradual decline in the plasma Gal-9 that was associated with the patient’s recovery (Fig. 1E). Based on the substantial difference between the magnitude of Gal-9 in the plasma of COVID-19 patients and in HCs, we hypothesized that the plasma Gal-9 level could be used as a surrogate marker to differentiate COVID-19 patients from HCs. Therefore, we used the receiver operating characteristic (ROC) curve to calculate the best cutoff value of the plasma Gal-9 to differentiate COVID-19 patients from HCs (39). The point of ROC curve related to the best sensitivity/specificity indicated that a level of plasma Gal-9 below 2,042 pg/ml separates HCs from COVID-19 patients with 94.96% sensitivity (95% confidence interval [CI] = 89.44% to 97.66%) and 94.92% specificity (95% CI: 86.08 to 98.61%) (Fig. 1F). The positive and negative predictive values of the test were 94.8% and 91.8%, respectively. However, when we analyzed the differential levels of Gal-9 in mild/moderate versus severe patients, the calculated area under the curve was 0.06091, which showed the lack of sensitivity to differentiate two patient groups (see Fig. S1A in the supplemental material). Besides, we noted that the plasma Gal-9 level was not associated with age (Fig. S1B). To determine the sensitivity of the plasma Gal-9 concentration in differentiating COVID-19 patients (*n* = 120) from HCs and those with either HIV or virus-associated solid tumors (*n* = 161), we applied a Gal-9 cutoff value of 2,042 pg/ml. These analyses revealed a sensitivity of 94.96% (95% CI: 89.44 to 97.67%) and specificity of 88.41% (95% CI: 82.61 to 92.46%) (Fig. 1G). Furthermore, we excluded HCs from our analysis, combined only virus-associated solid tumors and HIV-infected individuals (*n* = 103), and compared them to COVID-19 patients (*n* = 120) with the same cutoff value (2,042 pg/ml). This analysis indicated a sensitivity of 95% and specificity of 85.44% (Fig. 1H). Overall, our observations suggest that a cutoff value of 2,042 pg/ml for the plasma Gal-9 is a sensitive screening biomarker to discriminate COVID-19 patients from HCs.

10.1128/mBio.00384-21.1 FIG S1 (A) The receiver operating characteristic (ROC) curve showing the comparison between patients with moderate versus severe disease. (B) The plasma concentrations of Gal-9 are shown in COVID-19 patients based on age groups. (C) Representative flow plots of the gating strategy for NK cells. (D) Representative histogram plots and cumulative data of TNF-α expression in CD56^+^ CD16^−/lo^ NK cells. (E) Representative histogram plots and cumulative data of TNF-α expression in CD16^+^ CD56^−/lo^ NK cells. (F) Representative histogram plots and cumulative data of TNF-α expression in CD56^+^ CD16^+^ NK cells. Download FIG S1, TIF file, 1.2 MB. Copyright © 2021 Bozorgmehr et al. 2021 Bozorgmehr et al. https://creativecommons.org/licenses/by/4.0/ This content is distributed under the terms of the Creative Commons Attribution 4.0 International license.

### The plasma Gal-9 level is correlated with elevated proinflammatory cytokines and chemokines in COVID-19 patients.

Gal-9 can be secreted via an unknown mechanism(s) by different immune and nonimmune cells (29). Thus, we decided to determine if the peripheral blood mononuclear cells (PBMCs) or neutrophils from COVID-19 patients secrete Gal-9. PBMCs and neutrophils were cultured in the absence of any stimulation for 12 and 6 h *in vitro*, respectively. Then, culture supernatants were collected and subjected to Gal-9 quantification by enzyme-linked immunosorbent assay (ELISA). The 6-h culture was chosen due to the short lifespan of neutrophils compared to PBMCs. We found that PBMCs from COVID-19 patients with severe disease secreted a significantly higher quantity of Gal-9 compared to those with moderate disease and HCs (Fig. 1I). However, neutrophils from COVID-19 patients regardless of the disease status secreted higher levels of Gal-9 compared to their counterparts in HCs (Fig. 1J). These observations suggested that immune cells can be considered a potential source of soluble Gal-9 in the plasma of COVID-19 patients. Such a high level of Gal-9 in the plasma of COVID-19 patients, regardless of the source, highlights its potential stimulatory or inhibitory properties (40). Therefore, we decided to quantify a wide range of cytokines and chemokines in the plasma of COVID-19 patients to delineate the possible role of Gal-9 in the cytokine storm as reported elsewhere (38).

**FIG 2:**
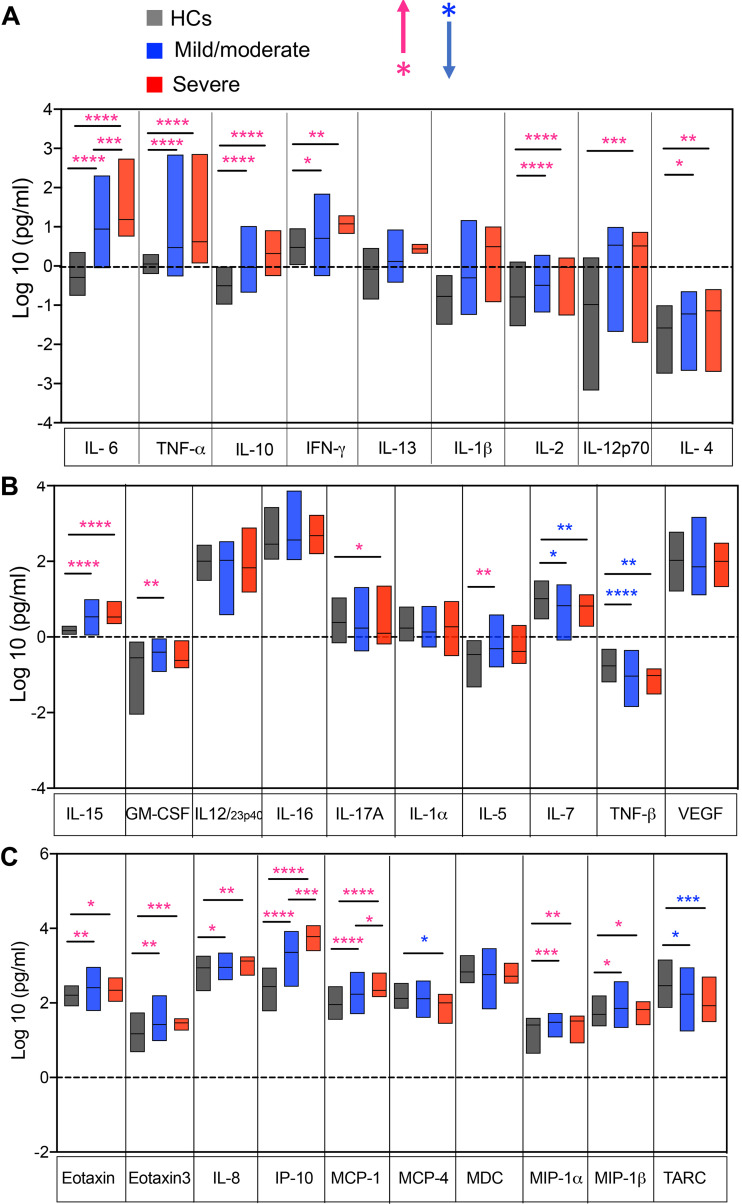
The elevation of a wide range of cytokines and chemokines in the plasma of COVID-19 patients. (A to C) Normalized and calculated concentrations of shown cytokines in the plasma of COVID-19 patients (*n* = 120) versus HCs (*n* = 59) as measured by Meso Scale Discovery (MSD). *, *P* < 0.05; **, *P* < 0.01; ***, *P* < 0.001; ****, *P* < 0.0001.

**FIG 3:**
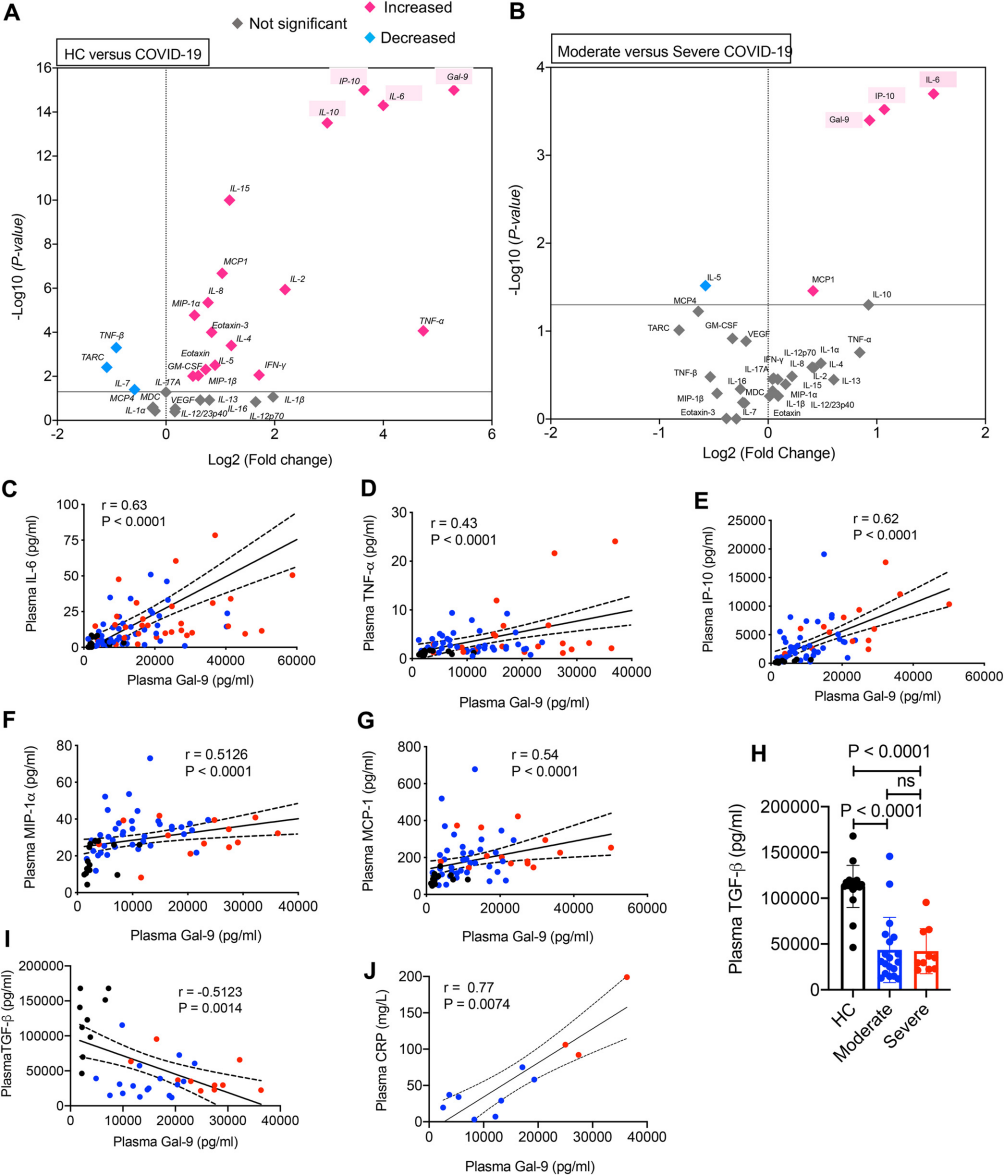
The correlation of plasma Gal-9 with proinflammatory cytokines and chemokines in COVID-19 patients. (A) Volcano plot illustrating the magnitude and significance of the differences in cytokine/chemokine serum concentrations in COVID-19 patients versus HCs. (B) Volcano plot illustrating the magnitude and significance of the differences in cytokine/chemokine plasma concentrations in COVID-19 patients with moderate versus severe disease. Diamonds marked in red and blue are significantly increased or decreased, respectively. The diamonds in gray show no significant difference. The significant difference in median plasma concentration was calculated for multiple testing. (C) Plasma concentrations of IL-6 show a significant positive correlation with plasma Gal-9 concentrations in COVID-19 patients. (D) Plasma concentrations of TNF-α show a significant positive correlation with plasma Gal-9 concentrations in COVID-19 patients. (E) Plasma concentrations of IP-10 show a significant positive correlation with plasma Gal-9 concentrations in COVID-19 patients. (F) Plasma concentrations of MIP-1α show a significant positive correlation with plasma Gal-9 concentrations in COVID-19 patients. (G) Plasma concentrations of MCP-1 show a significant positive correlation with plasma Gal-9 concentrations in COVID-19 patients. (H) Plasma concentration of TGF-β in HCs versus COVID-19 patients with either moderate or severe disease. (I) Plasma concentrations of TGF-β show a significant negative correlation with plasma Gal-9 concentrations in COVID-19 patients. (J) Plasma concentrations of CRP show a significant positive correlation with plasma Gal-9 concentrations in COVID-19 patients. Each point represents data from a patient. Bar, mean ± 1 standard error.

We observed significant elevation of a wide range of cytokines and chemokines including IL-6, TNF-α, IL-10, IFN-γ, IL-2, IL-4, IL-15, eotaxin, eotaxin3, IL-8, IP-10, MIP-1α, MCP-1, and MIP-1*β* in plasma specimens of COVID-19 patients (both mild/moderate and severe) versus HCs (Fig. 2A to C). Furthermore, we found that IL-6, IP-10, and MCP-1 concentrations were significantly higher in plasma specimens of patients with severe versus mild/moderate disease (Fig. 2A and C). While IL-17A was significantly higher in those with severe disease, IL-5 and GM-CSF were elevated in patients with mild/moderate disease (Fig. 2B). In contrast, TNF-β, IL-7, and thymus and activation regulated chemokine (TARC) were significantly downregulated in both patient groups but MCP-4 was significantly lower in severe patients versus HCs (Fig. 2B and C). However, we did not observe any difference in the plasma concentrations of IL-12/23p40, IL-16, vascular endothelial growth factor (VEGF), and macrophage derived chemokine (MDC) (Fig. 2B and C). A volcano plot of normalized data indicated that COVID-19 patients had a greater proinflammatory immune response as shown by the higher concentrations of multiple cytokines and chemokines (Fig. 3A). In particular, Gal-9, IL-6, IP-10, IL-10, and TNF-α were substantially higher in COVID-19 patients versus HCs (Fig. 3A). However, when the same analysis was performed on patients with mild/moderate versus severe disease, we found that only upregulation of IL-6, IP-10, Gal-9, and MCP-1 distinguished patients with severe from those with a mild/moderate disease (Fig. 3B). Our further analysis confirmed a positive correlation of the plasma Gal-9 concentration with IL-6, TNF-α, IP-10, MIP-1α, and MCP-1 in COVID-19 patients (Fig. 3C to G). In contrast, TGF-*β* levels were significantly lower in the plasma of COVID-19 patients compared to HCs, without any difference between patient groups (Fig. 3H), and thus, we found an inverse correlation between TGF-*β* and Gal-9 concentrations in plasma specimens of COVID-19 patients (Fig. 3I). Although we had C-reactive protein (CRP) data from a small number of patients, we noted a positive correlation of the plasma Gal-9 with CRP in those patients (Fig. 3J). These observations suggested a stimulatory role for Gal-9 in COVID-19 patients, possibly by enhancing innate immune cell activation (e.g., monocytes/macrophages, neutrophils, and NK cells) and induction of proinflammatory cytokines/chemokines.

### The rhGal-9 induces IL-6 and TNF-α secretion by monocytes and NK cells *in vitro*.

**FIG 4:**
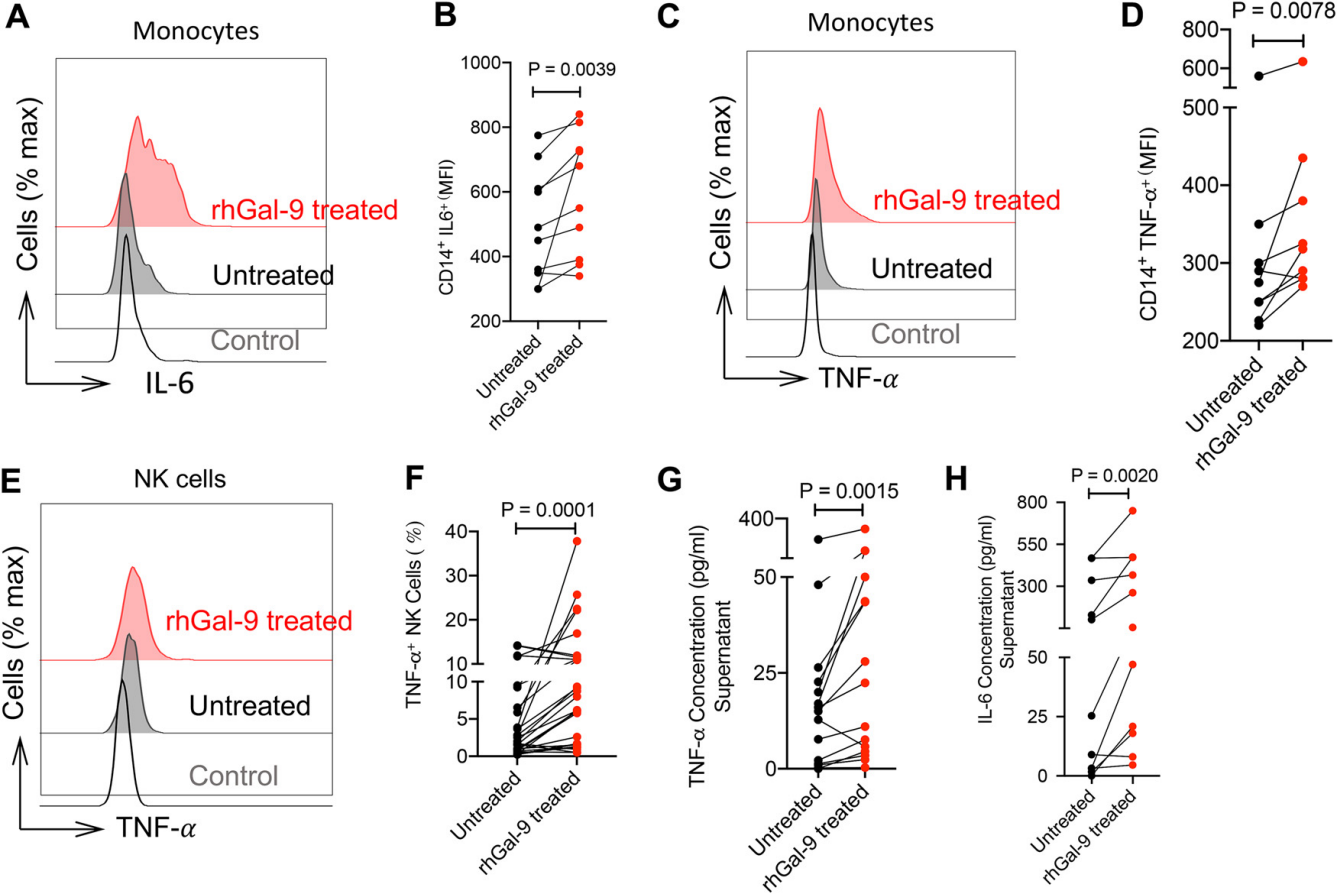
The rhGal-9 induces IL-6 and TNF-α secretion by monocytes and NK cells *in vitro.* (A and B) Representative histogram plots (A) and cumulative data (B) showing expression of IL-6 in monocytes from COVID-19 patients following *in vitro* stimulation with rhGal-9 (0.02 μg/ml) for 6 h as measured by the mean fluorescence intensity (MFI). (C and D) Representative histogram plots (C) and cumulative data (D) showing expression of TNF-α in monocytes from COVID-19 patients following *in vitro* stimulation with rhGal-9 (0.02 μg/ml) for 6 h. (E and F) Representative histogram plots (E) and cumulative data (F) showing percentages of TNF-α^+^ NK cells from COVID-19 patients following *in vitro* stimulation with rhGal-9 (0.02 μg/ml) for 6 h. (G and H) Culture supernatant concentrations of TNF-α (G) and IL-6 (H) in PBMCs of COVID-19 patients treated with rhGal-9 (0.02 μg/ml) for 12 h as quantified by ELISA. Each point represents data from a patient. Bar, mean ± 1 standard error.

Due to the high concentration of Gal-9 in the plasma of COVID-19 patients, we decided to investigate the effect of this protein on cytokine expression by monocytes and NK cells *in vitro*. For this purpose, we treated total PBMCs of COVID-19 patients with recombinant human Gal-9 (rhGal-9) (0.02 μg/ml), which mimics the mean detected Gal-9 level in the plasma, for 6 h. Then, IL-6 expression on monocytes and TNF-α in both monocytes and NK cells in comparison to nontreated cells were quantified. Interestingly, we observed a significantly higher IL-6 and TNF-α expression in Gal-9-treated compared to untreated monocytes (Fig. 4A to D). Similar observations were made for TNF-α expression in either total NK cells (Fig. 4E and F and Fig. S1C) or their subpopulations such as CD56^+^ CD16^lo/−^, CD16^+^ CD56^lo/−^, and CD56^+^ CD16^+^ NK cells (Fig. S1D to F). For verification in another set of experiments, we cultured total PBMCs with/without rhGal-9 (0.02 μg/ml) overnight. Then, we collected supernatants and measured IL-6 and TNF-α concentrations by ELISA. In agreement with our intracellular cytokine staining, we found that rhGal-9 treatment resulted in enhanced production of TNF-α and IL-6 by PBMCs (Fig. 4G and H). Taken together, these data indicate that the exogenous rhGal-9 exhibits a stimulatory function by enhancing the production of proinflammatory cytokines (e.g., IL-6 and TNF-α as the major monocyte-derived cytokines) in COVID-19 patients. Therefore, Gal-9 may contribute to the exacerbation of the cytokine storm in these patients.

### Distinct phenotypical characteristics of monocytes in COVID-19 patients.

**FIG 5:**
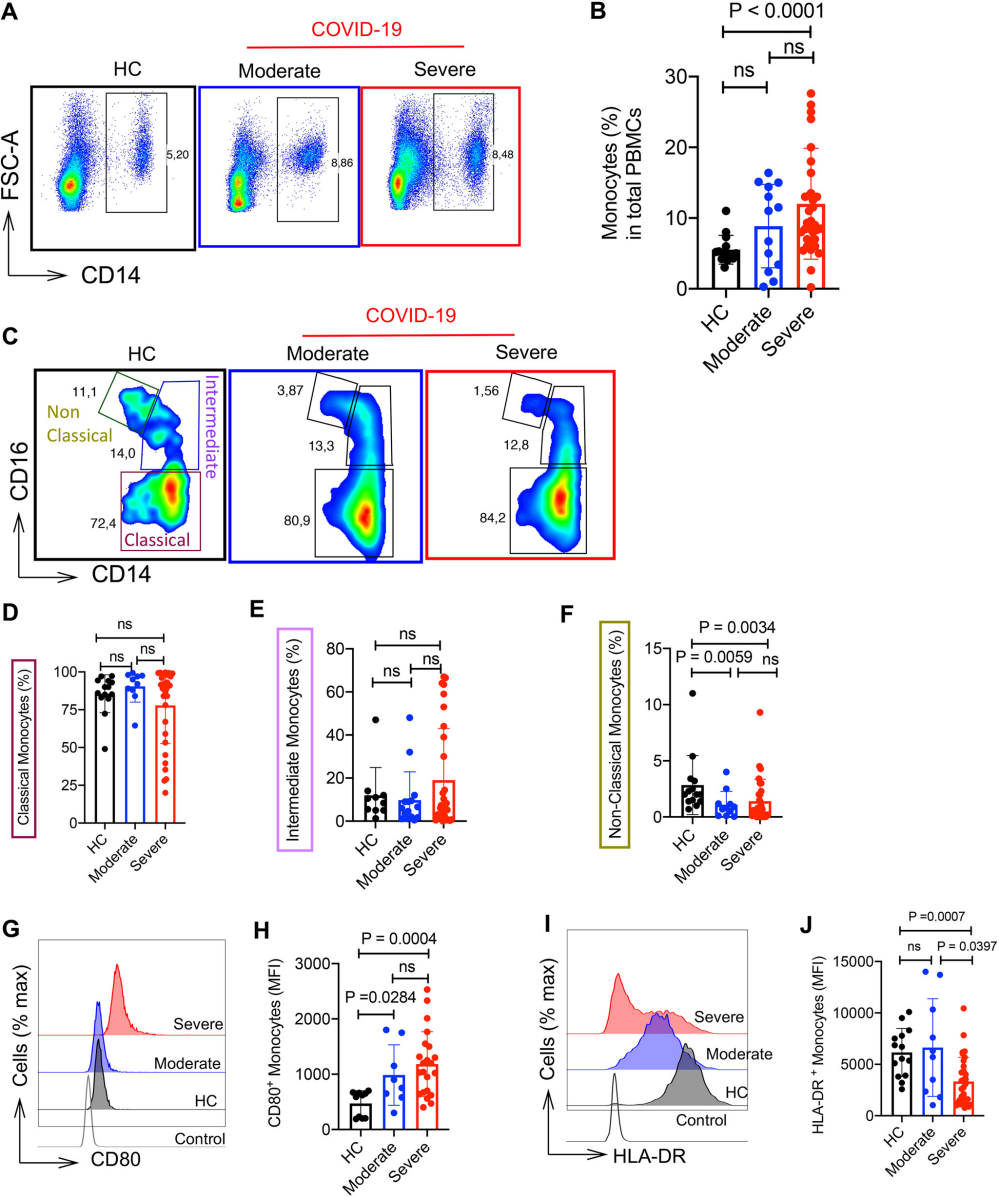
Monocytes are highly activated in COVID-19 patients. (A and B) Representative flow cytometry plots (A) and cumulative data (B) of percentages of monocytes in fresh PBMCs of HCs versus COVID-19 patients with either moderate or severe disease. (C to F) Representative flow cytometry plots (C) and cumulative data (D to F) of percentages of classical monocytes (CD14^+^ CD16^−/lo^) (D), intermediate monocytes (CD14^+^ CD16^+^) (E), and nonclassical monocytes (CD14^−/lo^ CD16^+^) (F) in fresh PBMCs of HCs versus COVID-19 patients with either moderate or severe disease. (G and H) Representative histogram (G) and cumulative data (H) of the mean fluorescence intensity (MFI) of CD80 expression on total monocytes from HCs compared to COVID-19 patients with moderate and severe disease. (I and J) Representative histogram (I) and cumulative data (J) of the MFI of HLA-DR expression on total monocytes from HCs compared to COVID-19 patients with moderate and severe disease. Each point represents data from a patient. Bar, mean ± 1 standard error.

Alteration in phenotype and functional properties of myeloid cells such as monocytes in SARS-CoV-2-infected individuals is well documented. In particular, recruited inflammatory monocytes in the lung are associated with disease progression (41). Thus, we analyzed the phenotype of monocytes in the peripheral blood of COVID-19 patients. We found a significant increase in the frequency of CD14^+^ monocytes in the severe group versus HCs. However, no difference was observed between the moderate and severe groups (Fig. 5A and B and Fig. S2A). Also, we analyzed the proportion of monocyte subpopulations among total monocytes as classical (CD14^hi^ CD16^−/lo^), intermediate (CD14^hi^ CD16^hi^), and nonclassical (CD14^−/lo^ CD16^hi^). However, we found no significant difference in the frequency of classical and intermediate monocytes between COVID-19 patients and HCs, and similarly between mild/moderate and severe groups (Fig. 5C to E). Of note, the frequency of the nonclassical monocyte subset was significantly lower in both COVID-19 patient groups compared to HCs (Fig. 5F). We further characterized their phenotype based on the expression of CD80 and HLA-DR. Our analysis showed a significantly higher intensity of CD80 expression on monocytes in COVID-19 patients compared to HCs without significant difference between patients with mild/moderate and those with severe disease (Fig. 5G and H). In contrast, the intensity of HLA-DR expression on monocytes was significantly lower in severe COVID-19 patients compared to the mild/moderate group and HCs (Fig. 5I and J). This was also reflected in the percentage of HLA-DR-expressing monocytes in COVID-19 patients compared to HCs (Fig. S2B and C), which is in agreement with other reports in sepsis (42) and COVID-19 patients (43). A light morphology examination of a blood smear from a COVID-19 patient confirmed an activated monocyte with intracytoplasmic vacuolization (Fig. S2D). Thus, the reduction in HLA-DR expression in COVID-19 patients favors a dysfunctional monocyte phenotype.

10.1128/mBio.00384-21.2 FIG S2 (A) Representative flow cytometry plots of the gating strategy for monocytes. (B and C) Representative flow cytometry plots (B) and cumulative data (C) of HLA-DR expression on monocytes in healthy controls (HC) versus COVID-19 patients with either moderate or severe disease. (D) A light morphology examination on a blood smear from a COVID-19 patient of an activated monocyte with intracytoplasmic vacuolization. (E) Representative flow cytometry plots of the gating strategy for neutrophils. (F and G) Representative flow cytometry plots (F) and cumulative data (G) of Gal-9 expression on unstimulated (unstim.) versus LPS (1 μg/ml)-stimulated neutrophils for 3 h. Download FIG S2, TIF file, 2.3 MB. Copyright © 2021 Bozorgmehr et al. 2021 Bozorgmehr et al. https://creativecommons.org/licenses/by/4.0/ This content is distributed under the terms of the Creative Commons Attribution 4.0 International license.

### Gal-9-expressing monocytes are highly activated.

**FIG 6:**
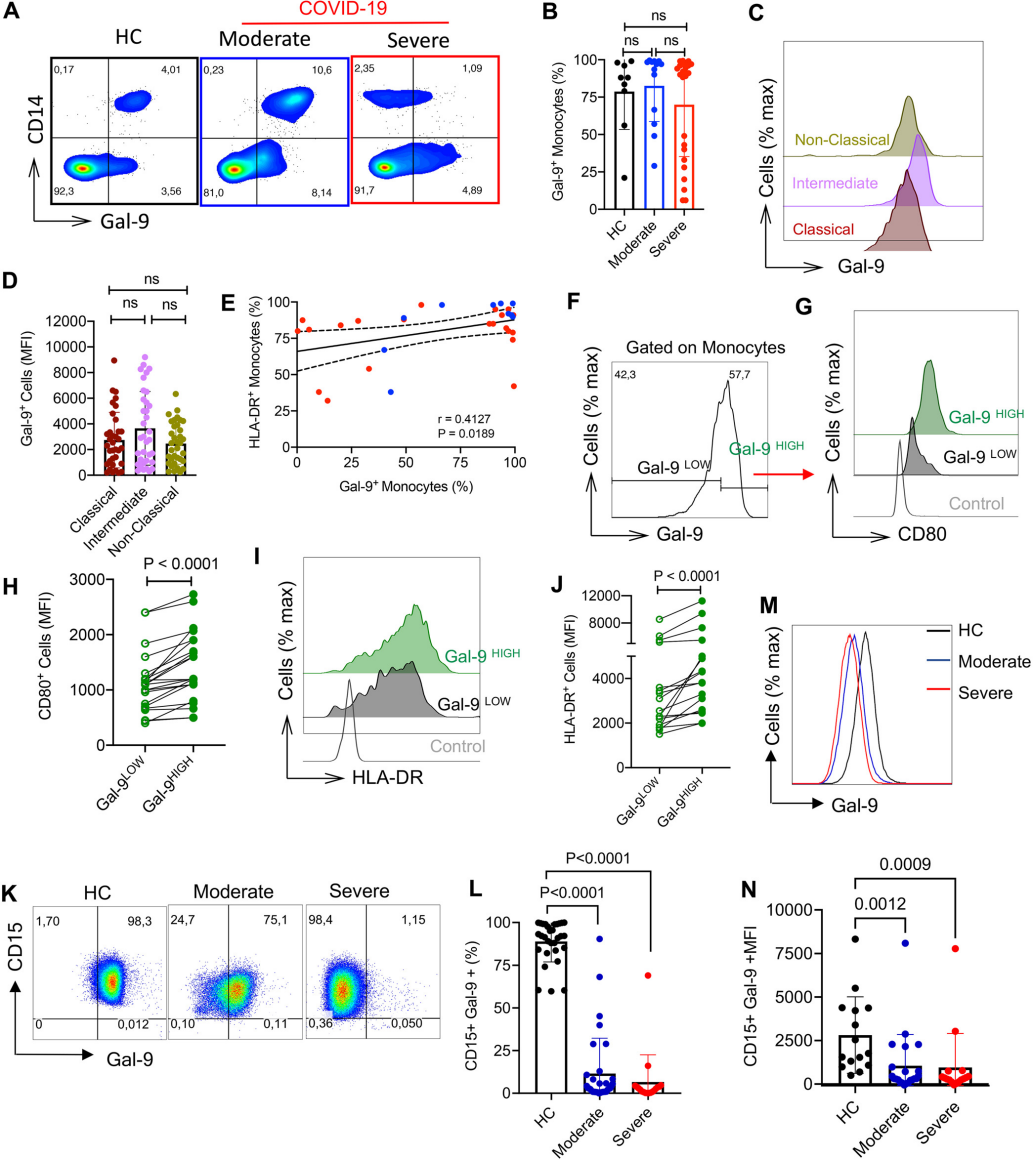
The higher intensity of surface Gal-9 expression is associated with activated monocytes, and activated neutrophils downregulate surface Gal-9. (A and B) Representative flow cytometry plots (A) and cumulative data (B) of percentages of Gal-9-expressing cells among total monocytes in HCs versus COVID-19 patients. (C and D) Representative histogram (C) and cumulative data (D) of the intensity of Gal-9 expression on different subpopulations of monocytes in COVID-19 patients. (E) Percentages of Gal-9^+^ monocytes show a significant positive correlation with percentages of HLA-DR^+^ monocytes in COVID-19 patients. (F) Representative histogram showing the intensity of Gal-9 expression in monocytes. (G and H) Representative histogram (G) and cumulative data (H) of the intensity of CD80 expression in Gal-9^lo^ versus Gal-9^hi^ monocytes in COVID-19 patients. (I and J) Representative histogram (I) and cumulative data (J) of the intensity of HLA-DR expression in Gal-9^lo^ versus Gal-9^hi^ monocytes in COVID-19 patients. (K and L) Representative flow cytometry plots (K) and cumulative data (L) of the percent Gal-9^+^ neutrophils coexpressing CD44 in HCs and COVID-19 patients. (M and N) Representative histogram (M) and cumulative data (N) of the intensity of Gal-9^+^ expression on neutrophils in HCs versus COVID-19 patients. Each point represents data from a patient. Bar, mean ± 1 standard error.

We analyzed the surface expression of Gal-9 on monocytes in COVID-19 patients to determine whether SARS-CoV-2 infection impacts Gal-9 expression in these cells. Our observations showed that although monocytes express fluctuating levels of Gal-9, we did not observe any significant difference in percentages of Gal-9^+^ monocytes between COVID-19 patients and HCs (Fig. 6A and B). Similarly, the intensity of Gal-9 expression remained unchanged among monocyte subsets (Fig. 6C and D). However, we found a positive correlation between the Gal-9- and HLA-DR-expressing monocytes (Fig. 6E). Furthermore, our observations confirmed that Gal-9^hi^ monocytes expressed significantly higher levels of CD80 and HLA-DR compared to their Gal-9^lo^ counterparts (Fig. 6F to J). These observations suggested that Gal-9^hi^ monocytes were more activated and stimulatory compared to their Gal-9^lo^ counterparts. Although percentages of Gal-9-expressing monocytes in COVID-19 and HC subjects were not significantly different, monocytes with greater intensity for Gal-9 expression (Gal-9^hi^) exhibited an activated phenotype in COVID-19 patients.

### COVID-19 infection induces Gal-9 downregulation in neutrophils.

We decided to delineate the impact of COVID-19 infection on Gal-9 expression on neutrophils, as the most abundant immune cells in the peripheral blood. We found that SARS-CoV-2 infection results in significant downregulation of surface Gal-9, which was evident not only in percentages of Gal-9^+^ neutrophils (Fig. 6K and L and Fig. S2E) but also in the intensity of Gal-9 expression in neutrophils from COVID-19 patients versus HCs (Fig. 6M and N). To better understand the mechanism underlying Gal-9 downregulation in neutrophils, we measured Gal-9 expression levels on neutrophils from HCs following stimulation with lipopolysaccharide (LPS) for 3 h. We found that neutrophil activation was associated with the downregulation of Gal-9 (Fig. S2F and G). These data suggest that activation of neutrophils following SARS-CoV-2 infection results in Gal-9 shedding from neutrophils as we have shown in HIV infection (G. Dunsmore, E. Perez Rosero, S. Shahbaz, M. D. Santer, J. Jovel, P. Lacy, S. Houston, and S. Elahi, submitted for publication). Thus, activated neutrophils may serve as one potential source of the plasma Gal-9 in COVID-19 infection.

## DISCUSSION

Since the elevation of plasma Gal-9 in chronic and acute viral infections has been reported (19), we asked whether this was the case for COVID-19 infection. We found extremely elevated levels of plasma Gal-9 in COVID-19 patients, which was more pronounced in those with severe disease. The plasma Gal-9 concentration in COVID-19 patients surpasses the detectable levels reported in other conditions such as HIV, dengue fever, influenza, and virus-associated solid tumors (19, 22, 24). Based on our findings, we hypothesized that the plasma Gal-9 concentration is a diagnostic biomarker that can differentiate non-COVID-19 from COVID-19 patients as suggested for HIV (44). By using the ROC curve (45), we found that a cutoff value of 2,042 pg/ml can discriminate COVID-19 patients from either HCs or patients with HIV and virus-associated cancers. It is worth noting that most HCs have low to undetectable Gal-9 levels. The diagnostic power was calculated based on the ELISA kit from R&D Systems, which might be different from other ELISA kits (46). For instance, it has been reported that the full-length Gal-9 (F-Gal-9) levels were extremely elevated in dengue fever (38). However, the cleaved/truncated from of Gal-9 (T-Gal-9) was considered a more sensitive biomarker to distinguish HIV-infected individuals from those coinfected with tuberculosis (47). Therefore, it would be worth examining F-Gal-9 versus T-Gal-9 levels in COVID-19 patients since the R&D Systems ELISA kit quantifies both forms of Gal-9. Although the diagnostic power of the plasma Gal-9 ELISA was not sufficient to distinguish mild/moderate from severe COVID-19 infection, its high sensitivity and specificity (∼95%) make it an attractive point-of-care test (48) or a rapid diagnostic tool for mass screening (49). RT-PCR assays with sensitivity and specificity of greater than 95% are the operational gold standard for detecting SARS-CoV-2 in clinical settings (50). Although no single gold standard diagnostic test exists and because of hidden issues associated with false-positive and -negative RT-PCR results, verification by other complementary and inexpensive tests such as the plasma Gal-9 assay should be considered. However, due to the reduced specificity of this test in patients with underlying conditions (e.g., HIV and cancer), additional confirmatory tests such as viral RNA detection should be taken into consideration. The main question is the biological nature underlying the elevation of Gal-9 in COVID-19 patients. Gal-9 is widely expressed intracellularly in a variety of immune and nonimmune cells including lung epithelial cells and endothelial cells (19). Furthermore, increased Gal-9 in bronchoalveolar lavage samples of virus-infected animals has been reported (51). Similarly, human cytomegalovirus infection enhances the release of Gal-9 by human foreskin fibroblasts (52). Therefore, we assume that an elevation in the plasma Gal-9 in COVID-19 infection may be caused by the drastic viral infection or cell-associated damage/lysis resulting in its leakage from the cytoplasm. It is reported that Gal-9 gets recruited into immune synapses upon T cell activation, and thus, intracellular Gal-9 acts as a regulator of T cell activation (53). In agreement, we have reported the translocation of intracellular Gal-9 in activated T cells in HIV and cancer (24, 25). These observations suggest that activated immune cells may serve as a potential source of Gal-9 translocated into the plasma of COVID-19 patients. In agreement, we detected a substantial quantity of Gal-9 in the culture supernatants of PBMCs and neutrophils of COVID-19 patients *in vitro*. This might be related to their hyperimmune activation status or infection-associated indirect cell damage. Gal-9 is reported to be degraded swiftly by proteinases (47). This might be a protective mechanism to prevent off-target effects of this immunomodulatory protein. Gal-9 can exhibit either inhibitory or stimulatory effects depending on the corresponding ligand (e.g., PDI, TIM-3, CD44, CD137, etc.). Considering the well-documented cytokine storm in COVID-19 infection, it is less likely that the plasma Gal-9 can exhibit any inhibitory effects such as suppression of Th1 immunity (54) and promotion of regulatory T cells (Tregs) (55). Gal-9 via interaction with CD44 requires synergistic effects of TGF-β to enforce induced Treg differentiation, which is less likely to occur in COVID-19 patients with significant downregulation of TGF-β levels. Another possibility is the differential effect of the F-Gal-9 versus T-Gal-9. For example, F-Gal-9 can be cleaved by MMP9, which is highly increased in the plasma of COVID-19 patients (56), resulting in the abundance of T-Gal-9 in COVID-19 patients. On the other hand, the impact of Gal-9 on the infectivity of SARS-CoV-2 is unclear. Gal-9 plasma levels reflect viral load in acute dengue virus infection (38), and it has been reported that Gal-9 via interaction with PDI enhances HIV infection (57). In contrast, Gal-9 via interaction with TIM-3 can suppress the infectivity of CD4^+^ T cells to HIV infection (22).

**FIG 7:**
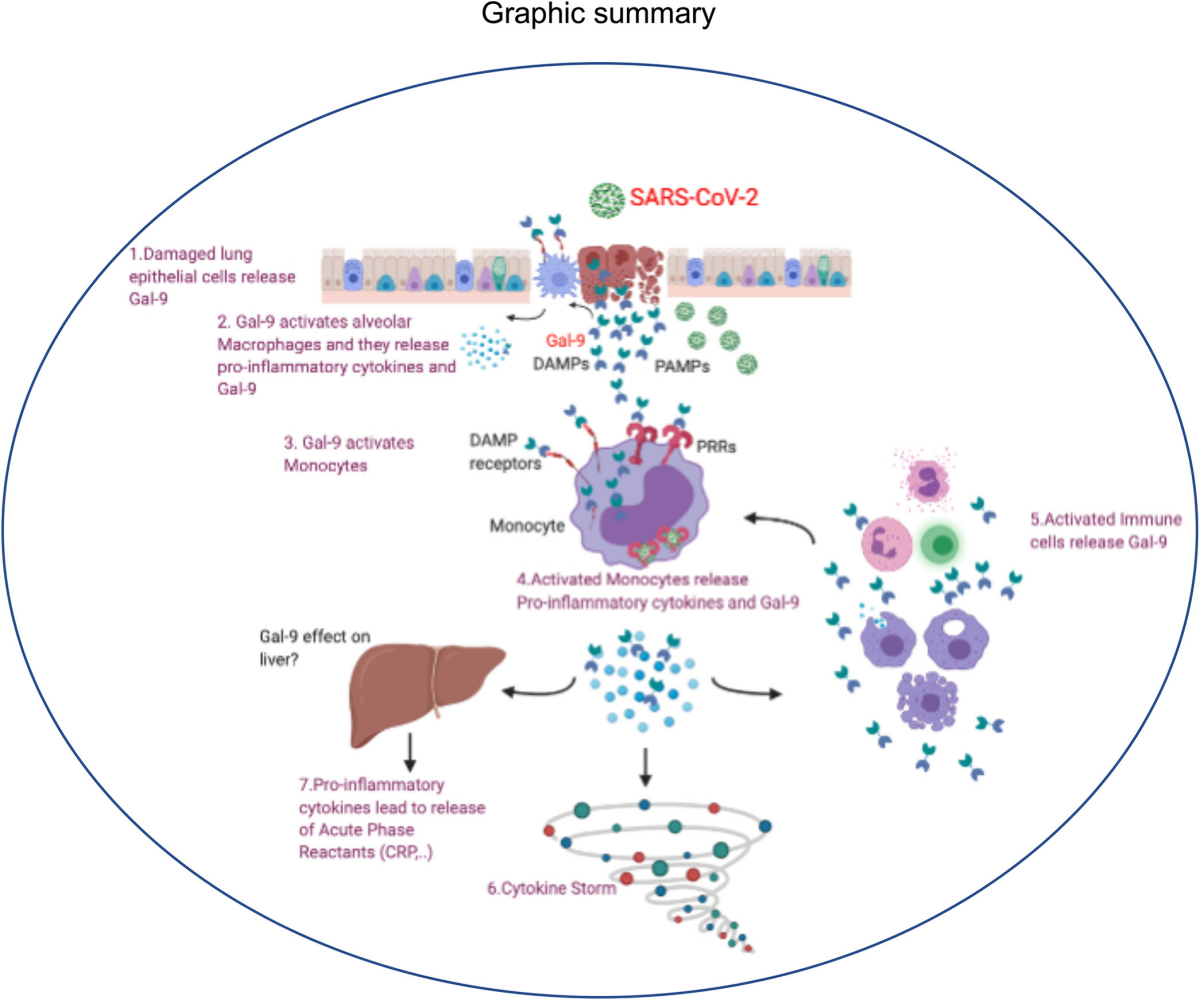
The model shown envisions how Gal-9 activates innate immune cells contributing to the cytokine storm. The damaged lung epithelial cells following SARS-CoV-2 infection release Gal-9, which activates alveolar macrophages, resulting in the secretion of proinflammatory cytokines and Gal-9 from the activated or apoptotic cells. Subsequently, Gal-9 activates monocytes and other immune cells, orchestrating another wave of proinflammatory cytokines and Gal-9 release. These cascades of events may also impact liver function (e.g., CRP), exacerbating the cytokine storm.

Broadly speaking, cytokine storm designates a hyperactive immune response characterized by the presence of cytokines, chemokines, and several other mediators as part of a well-conserved innate immune system to clear the virus (58). In agreement, we observed significant elevation of a wide range of proinflammatory cytokines and chemokines in the plasma of COVID-19 patients compared to HCs as reported by others (58–60). Our further analysis showed a strong correlation of the plasma Gal-9 levels with prominent mediators of cytokine storm (IL-6, TNF-α, MCP-1, IP-10, and MIP-1α) and CRP as reported in dengue fever (38). Therefore, our data suggest that the massive level of plasma Gal-9 in COVID-19 patients serves as a DAMP (34) that exerts its actions on several immune cells such as monocytes/macrophages, NK cells, and neutrophils to exacerbate cytokine storm. In agreement, we found induction of IL-6 and TNF-α by rhGal-9 in PBMCs, in particular, monocytes and NK cells, which is consistent with other reports (61, 62). The concentration of Gal-9 needed to evoke these responses was far lower (2,000 pg/ml) than the highest concentration of Gal-9 observed in some patients (>100,000 pg/ml). Thus, the debate on the role of Gal-9 should be based on the assumption that the local concentration of this lectin at the site of infection/injury is extremely high. Such a massive Gal-9 level may cause cell death in immune and nonimmune cells (63, 64) by a different mechanism(s) such as atypical ubiquitination (65). Subsequently, cell death causes leakage of the cytoplasm Gal-9, causing a vicious cycle. It is worth noting that lower IL-7 in COVID-19 patients may compromise adaptive immunity since it plays an important role in the maintenance of mature T cells, and it also contributes to B cell development (66). Moreover, higher IL-17A in COVID-19 patients with severe disease supports the potential role for this cytokine in COVID-19-related acute respiratory distress syndrome as reported elsewhere (67). Taken together, our study confirmed the massive elevation of Gal-9 in the plasma of COVID-19 patients regardless of the disease status. Based on our findings, we propose a complex and versatile role for this lectin in the acute phase of COVID-19 infection and the cytokine release syndrome (Fig. 7). Therefore, further studies in larger cohorts are needed to confirm our proposed diagnostic potential of the plasma Gal-9 as a sensitive biomarker in COVID-19 patients. More importantly, understanding the underlying mechanism(s) of Gal-9 release and/or its possible neutralization merits further investigations.

Of note, monocyte dysregulation is reported in COVID-19 patients (68); however, it is unclear whether Gal-9 can play a role in this dysregulation process. What we observed was an association between an activated monocyte phenotype (CD80^+^ HLA-DR^+^) and higher surface Gal-9 expression, which may suggest a role for Gal-9 in the exacerbation of cytokine production by monocytes. In addition to the cytokine release syndrome, CD146^+^ circulating endothelial cells are increased in COVID-19 patients (69). Since the interaction between Gal-9 and CD146^+^ contributes to the aggregation of infected red blood cells and lymphocytes in cerebral malaria (70), Gal-9 may play a role in coagulopathy in COVID-19 patients. Nevertheless, the potential role of massive plasma Gal-9 in coagulopathy and thrombotic phenomena in COVID-19 patients warrants further investigations.

We are aware of our study limitations. For example, we did not have access to bronchoalveolar lavage to quantify the concentration of Gal-9 at the site of infection/inflammation and whether alveolar macrophages express higher or lower levels of Gal-9. Although Gal-9 may be proposed as a viable diagnostic test in screening symptomatic patients, one of the limitations of this study is that there was no control group which was comprised of symptomatic patients with other bacterial, influenza virus, rhinovirus, or metapneumovirus infections. So, based on the findings in this study it may be a useful test only in symptomatic patients. Although we did not observe a significant difference in the plasma Gal-9 levels between patients with mild and moderate disease, larger patient cohorts are needed to confirm our findings, in particular in asymptomatic individuals. We were unable to perform experiments with animal models to better understand the role of Gal-9 in cytokine storm *in vivo* with the potential therapeutic implication of Gal-9 neutralization/inhibition approaches. It is worth mentioning that since we have used fresh blood samples for our studies, our data may differ from some published data on the frequency/phenotype of monocyte subpopulations since the cryopreservation process can impact the viability/functionality of antigen-presenting cells such as monocytes. We believe it would be crucial to include Gal-9 in a predictive model of COVID-19 patients and mortality since several large cohort studies have demonstrated that D-dimer and IL-6 in addition to clinical factors predict mortality in hospitalized COVID-19 patients.

## MATERIALS AND METHODS

### Study population.

Blood samples were collected from 146 COVID-19 patients for this noninterventional study from different hospitals in Edmonton, Canada. Patients were SARS-CoV-2^+^ PCR confirmed for SARS-CoV-2. Sixty-one were critically ill patients admitted to the Intensive Care Unit (ICU), whom we defined as having severe disease. Of the remaining patients, 80 were hospitalized on a hospital ward with moderate disease (see Table S1 in the supplemental material) and 5 patients without hospitalization were considered patients with mild disease. For comparison, we recruited 59 healthy individuals without clinical symptoms, recent travel, respiratory infections, or exposures to COVID-19 patients. In addition, these individuals were HIV, hepatitis C virus (HCV), and hepatitis B virus (HB) seronegative. We did not observe any association between the Gal-9 levels and the type of treatment (Fig. 1E). It appears Gal-9 naturally declines as patients recover.

10.1128/mBio.00384-21.3 TABLE S1 Patient demographic information and laboratory values. Download Table S1, PDF file, 0.1 MB. Copyright © 2021 Bozorgmehr et al. 2021 Bozorgmehr et al. https://creativecommons.org/licenses/by/4.0/ This content is distributed under the terms of the Creative Commons Attribution 4.0 International license.

### Ethics statement.

This study was approved by the Human Research Ethics Board (HREB) at the University of Alberta (Pro00099502). Waiver of consent was obtained by the HREB for those patients admitted to the Intensive Care Unit (ICU), but a verbal consent was required from all other patients. However, written consent was obtained from 5 mild cases who did not require hospitalization (HREB no. Pro00100207). Also, the HREB approved blood collection from HCs (Pro00063463). Written informed consent was obtained from HCs.

### Sample collection and processing.

The plasma was collected from the fresh EDTA blood samples and stored at −80°C for further analysis. Then, the peripheral blood mononuclear cells (PBMCs) were isolated using the Ficoll-Paque gradient method (GE Healthcare).

The red blood cell pellet containing neutrophils was removed, and red blood cells were lysed using a red blood cell lysis buffer (0.155 M NH_4_Cl, 10 mM KHCO_3_, and 0.1 mM EDTA). Freshly isolated polymorphonuclear cells were washed with 4°C phosphate-buffered saline (PBS) and put in culture medium (RPMI 1640; Sigma) supplemented with 10% fetal bovine serum and antibiotics (Sigma). Neutrophils were stimulated for 1 h at 37°C *in vitro* by culturing 1 × 10^6^ polymorphonuclear leukocytes (PMNs) in the presence or absence of lipopolysaccharide (LPS) at 1 μg/ml.

### Flow cytometry.

The fluorochrome-conjugated antibodies were purchased from ThermoFisher Scientific, BD Biosciences, or BioLegend, including anti-CD3 (SK7), anti-CD19 (HIB19), anti-CD14 (M5E2), anti-CD16 (B73.1), anti-CD15 (HI98), anti-CD56 (B159), anti-Gal-9 (9M1-3), anti-CD80 (L307.4), anti-CD44 (515), anti-HLA-DR (G46-6), anti-TNF-α (MAB11), and anti-IL-6 (MQ2-13A5). The LIVE/DEAD kit (Life Technologies) was used to assess cell viability. Stained cells were fixed in paraformaldehyde (PFA, 4%), and data were acquired on a Fortessa-X20 or LSR Fortessa-SORP flow cytometer (BD Biosciences). Data were analyzed using Flow Jo software (version 10).

### Cell culture and *ex vivo* cytokine measurement.

In some experiments, 1 × 10^6^ human PBMCs from COVID-19 patients were cultured with or without 0.02 μg/ml of the recombinant human Galectin-9 (rhGal-9; Gal-Pharma, Japan) in the presence of the protein transport inhibitor brefeldin-A (BD Biosciences) for 6 h. Next, cells were subjected to surface staining for NK or monocyte markers depending on the experiment followed by fixation and permeabilization (BD Cytofix/Cytoperm) and intracellular cytokine staining (ICS) for IL-6 and TNF-α according to our protocols (71). Then cells were acquired on a Fortessa-X20 or LSR Fortessa-SORP flow cytometry (BD Biosciences). Data were analyzed using Flow Jo software (version 10).

For cytokine measurement in culture supernatants, 1 × 10^6^ human PBMCs were cultured in the absence or presence of rhGal-9 (0.02 μg/ml) for 12 h. Then, culture supernatants were collected for measuring cytokines (TNF-α [DY210-05] and IL-6 [DY206-05]) by ELISA (R&D Systems).

### Cytokine and chemokine analysis.

Plasma samples were centrifuged for 15 min at 1,500 × *g* followed by dilution at 2- and 4-fold for quantifying cytokine and chemokine profiles, respectively. Concentrations of cytokines and chemokines were quantified using the V-plex Plus proinflammatory panel 1, cytokine panel 1 kit, and chemokine panel 1 kits from Meso Scale Discovery (MSD) according to the manufacturer’s instruction, which included IFN-γ, IL-1*β*, IL-2, IL-4, IL-6, IL-8, IL-10, IL-12p70, IL-13, TNF-α, GM-CSF, IL-1α, IL-5, IL-7, IL-12/23p40, IL-15, IL-16, IL-17A, TNF-α, VEGF, eotaxin, MIP-1α, eotaxin-3, TARC, IP-10, MIP-1*β*, MCP-1, MDC, and MCP-4. A total of 120 plasma samples from COVID-19 patients and 59 plasma samples from healthy subjects were examined for cytokine/chemokine analysis. Data were acquired on the V-plex Sector Imager 2400 plate reader. Analyte concentrations were extrapolated from a standard curve calculated using a four-parameter logistic fit using MSD Workbench 3.0 software. CRP concentrations were received from the patient’s clinical files. The plasma Gal-9 was quantified using ELISA (R&D; DY 2045) as we reported elsewhere (24, 25).

### Statistical analysis.

Statistical analysis was performed using GraphPad Prism 9 (GraphPad Software, Inc.). Mann-Whitney U tests or Wilcoxon signed-rank tests were used to compare data sets that were nonpaired or paired, respectively. Data were presented as means and standard deviations (mean ± SD). When more than two groups were compared, one-way analysis of variance (ANOVA) followed by Tukey’s test was used to compare the results. Correlation analysis was performed by nonparametric Spearman correlation.
