# Supplementary figures and images for: Galectin-9, a Player in Cytokine Release Syndrome and a Surrogate Diagnostic Biomarker in SARS-CoV-2 Infection
Source: mBio. 2021 May 4;12(3):e00384-21. doi: 10.1128/mBio.00384-21 (PMC8262904; doi:10.1128/mBio.00384-21)

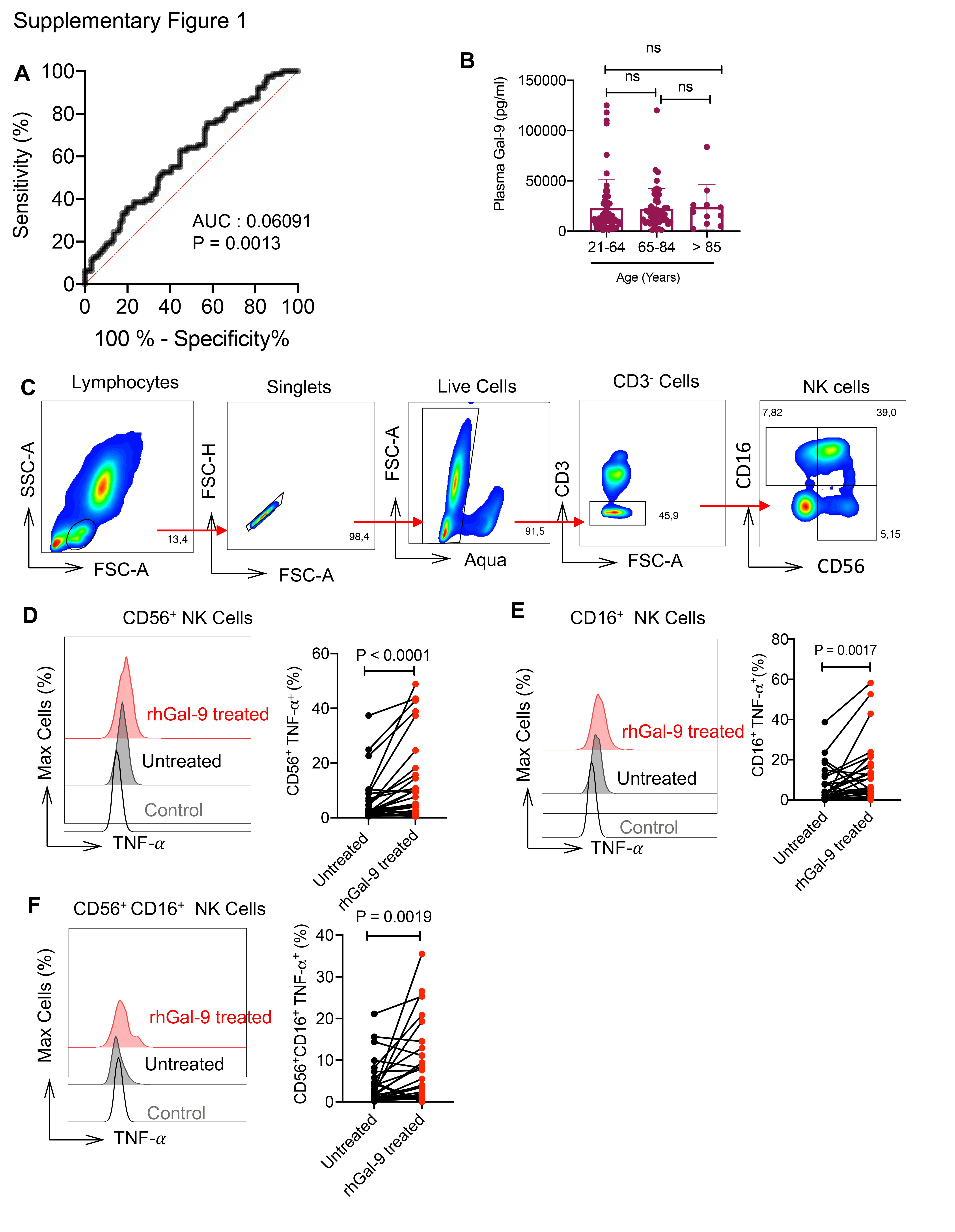

Supplement: FIG S1 [file mbio.00384-21-sf001.tif]

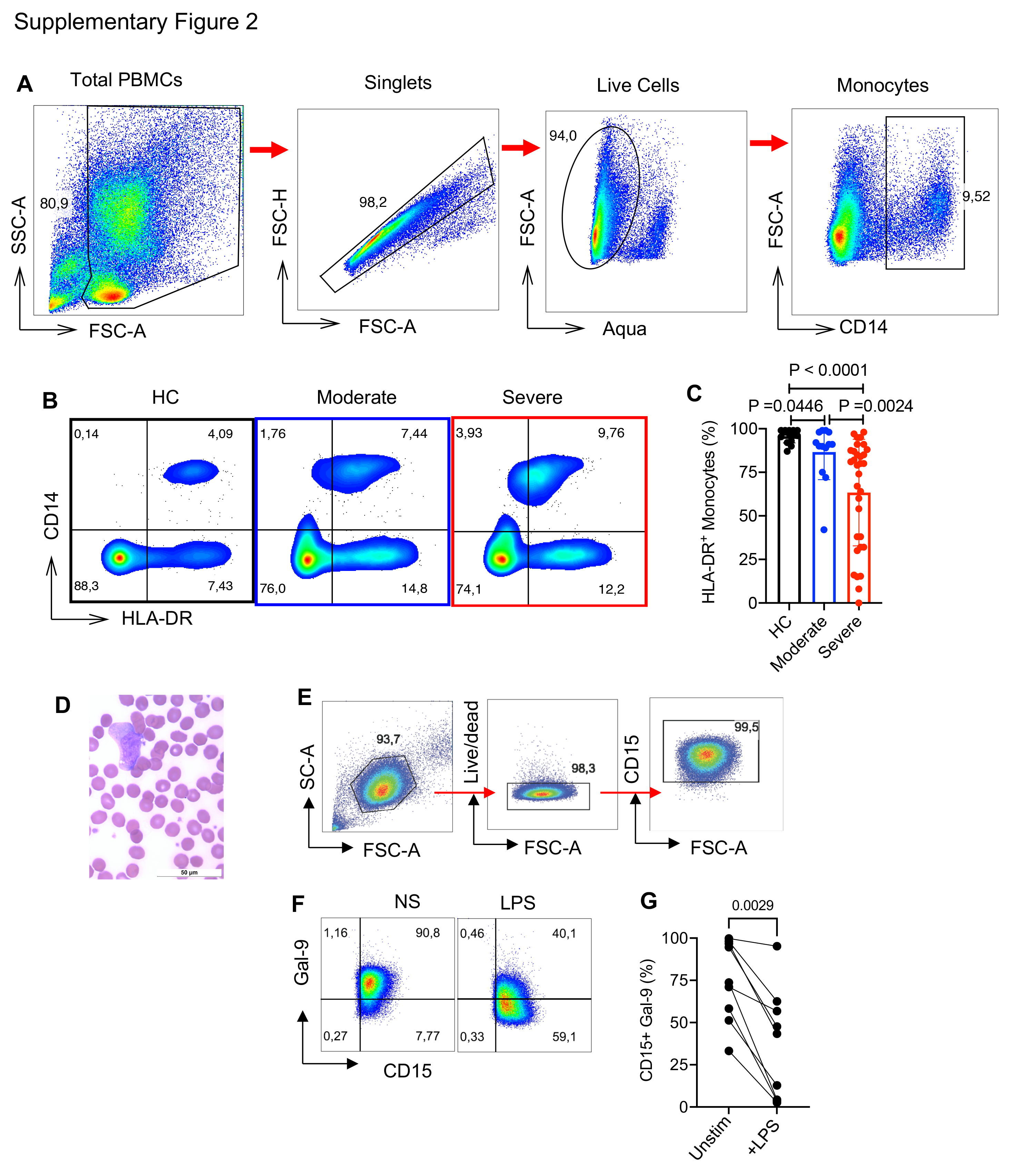

Supplement: FIG S2 [file mbio.00384-21-sf002.tif]
