# Supplementary material for: Galectin-9, a Player in Cytokine Release Syndrome and a Surrogate Diagnostic Biomarker in SARS-CoV-2 Infection
Source: mBio. 2021 May 4;12(3):e00384-21. doi: 10.1128/mBio.00384-21 (PMC8262904; doi:10.1128/mBio.00384-21)
Supplement: TABLE S1 [file mbio.00384-21-st001.pdf]

Supplementary Table 1. Characteristics of the patients.

Characteristics	All Patients (N=146)	Mild/Moderate (N=85, 58%)	Severe (N=61, 42%)	P-Value
Baseline and Demographic				
Median Age-yr	67(61-68)	68 (61-70)	67 (58-68)	0.381
Male-no. (%)	86 (59%)	49 (57%)	37 (43%)	0.07
Median Laboratory Values				
Creatinine (IU/L)		90.71	130.61	
WBC count (x10^9/L)		7.56	7.74	0.848
Neutrophils (x10^9/L)		5.66	6.47	0.957
Lymphocytes (x10^9/L)		1.2	0.68	0.003
Hemoglobin (g/L)		130	132	0.400
CPK (IU/L)		211.86	431.12	0.736
LDH (IU/L)		286.26	389.68	0.156
Troponin I ($\mu g/ml$)		0.05	1.57	0.003
D-Dimer ($\mu g/ml$)		1.44	1.75	0.010
Ferritin ($\mu g/L$)		631	1177	0.031
CRP (mg/L)		73.5	165	0.010

P value is comparing patients admitted to the ICU/severe versus mild/moderate patients. WBC (white blood cells), CRP (C-reactive protein), CPK (Creatine phosphokinase), LDH (Lactate dehydrogenase).
